# Competency Gradients in Advanced Practice Nurses, Specialist Nurses, and Registered Nurses: A Multicentre Cross-Sectional Study

**DOI:** 10.3390/ijerph19148415

**Published:** 2022-07-09

**Authors:** Laura Gutiérrez-Rodríguez, Silvia García-Mayor, Álvaro León-Campos, Alberto José Gómez-González, Bibiana Pérez-Ardanaz, Susana Rodríguez-Gómez, Marta Fajardo-Samper, Juan Carlos Morilla-Herrera, José Miguel Morales-Asencio

**Affiliations:** 1 Department of Nursing, Faculty of Health Sciences, University of Málaga, 29071 Málaga, Spain; laura_gr@uma.es (L.G.-R.); sgmayor@uma.es (S.G.-M.); gomezgonzalez88@uma.es (A.J.G.-G.); bibianap@uma.es (B.P.-A.); jmorilla29@uma.es (J.C.M.-H.); jmmasen@uma.es (J.M.M.-A.); 2 Biomedical Research Institute of Málaga (IBIMA), 29010 Málaga, Spain; 3 Regional Ministry of Health of Andalusia (CSJA), 41020 Sevilla, Spain; susana.rodriguez.gomez@gmail.com; 4 Sant Joan de Déu Barcelona Children’s Hospital, 08950 Barcelona, Spain; marta.fajardo@sjd.es

**Keywords:** advanced practice, nursing roles, leadership, professional regulation

## Abstract

(1) Background: Identifying differences in the competencies of different areas of nursing is a crucial aspect for determining the scope of practice. This would facilitate the creation of a formal structure for clinical practice in advanced and specialised services. The aims of this study are to analyse the distribution of advanced competencies in registered, specialist and advanced practice nurses in Spain, and to determine the level of complexity of the patients attended by these nurses. (2) Methods: A cross-sectional study was developed on registered, specialist and advanced practice nurses, all of whom completed an online survey on their perceived level of advanced competencies and their professional characteristics. (3) Results: In total, 1270 nurses completed the survey. Advanced practice nurses recorded the highest self-perceived level of competency, especially for the dimensions of evidence-based practice, autonomy, leadership and care management. (4) Conclusions: Among registered, specialist and advanced practice nurses, there are significant differences in the level of self-perceived competencies. Patients attended by advanced practice nurses presented the highest levels of complexity. Understanding these differences could facilitate the creation of a regulatory framework for clinical practice in advanced and specialized services.

## 1. Introduction

In recent years, many health systems have had to face the challenges arising from epidemiological and demographic changes, such as increasing costs, clinical variability, quality considerations, the creation of new health policies and programmes, health inequalities, population ageing (with the consequent increase in life expectancy) and chronic diseases [1]. Against this backdrop, the nursing profession is called upon to contribute to health care reform and to help meet the demands for a quality, safe, accessible and patient-centred health care system [2].

In order to cope with this scenario, new nursing roles have emerged, such as the advanced practice nurse (APN) [3], who is defined by the International Council of Nurses (ICN) as “a registered nurse (RN) who has acquired the expert knowledge base, complex decision-making skills and clinical competencies for expanded practice, the characteristics of which are shaped by the context and/or country in which s/he is credentialed to practice. A master’s degree is recommended for entry level” [4] (p. 9). Since the inception of this professional figure, APNs have provided quality, effective and safe care to a wide range of patients, in multiple settings [5]. One such is the care of chronically ill persons, which is often provided by the case management nurse (CMN) [6].

In conjunction with the development of the APN, another important role is played by the specialist nurse (SN), a nursing professional who is more highly qualified than the RN and has specialist knowledge in a specific area of nursing [7].

The existence of different nursing roles and levels of competency provides the health service with appropriate means to address the diverse challenges that may arise. However, the lack of consensus in defining the roles and competencies of APNs and SNs, together with the absence of regulatory frameworks in this respect, can result in the terms being used ambiguously [8].

In their analysis of nursing specialities in Europe, Ranchal et al. [9] observed heterogeneity in terms of training programmes, levels of certification, regulation and scope of practice. These divergences and lack of clarity also exist in the case of advanced practice, where there is still debate about concepts, roles and competencies [10]. Some authors have addressed these questions [3,11,12,13,14], but there is still no global consensus, and disagreements may even arise within a single country [15,16]. Nonetheless, the absence of a solid conceptual and regulatory framework does not prevent different countries from implementing these services in different health care settings. In Spain, this conceptual confusion may be more noticeable than elsewhere, due to the confluence of several distinguishing factors. Firstly, the decentralised nature of government in Spain, with 17 largely autonomous regions (termed Autonomous Communities), means that each of the 17 health systems enjoys a high level of independence in organising the provision of services and prioritising health policies [17]. Furthermore, within each Autonomous Community different organisations exist to accredit professional competencies; the categories established may receive different levels of remuneration, but these differences do not necessarily imply any modification of clinical roles and responsibilities. This further hinders the implementation of these roles and contributes to confusion [18]. Finally, the Spanish training system for nursing specialities is based on a two-year clinical residency programme (which is inspired in the medical residency training programme), after a national examination [19]. For SNs, this system addresses six clinical specialties: Midwifery, Mental Health Nursing, Geriatric Nursing, Occupational Health Nursing, Community Nursing and Paediatric Nursing [20]. However, the residency programme does not imply that these nurses necessarily develop advanced nursing roles, or even as specialists. This is one of the main confounding factors in comparison with other countries, where the Clinical Nurse Specialist (CNS) is one of the most widely recognised roles in advanced practice [21]. Similar incongruities have been described in another countries [22,23].

In view of these considerations, we believe it necessary to stipulate the differences in competencies between the different types of nurses in order to clarify and distinguish the areas of advanced practice and specialisation, and their coordination with the work of the RN. The classical conceptual framework of Daly and Carnwell [24], developed in the Anglo-Saxon context, highlighted the need to define these areas, and the concepts of extension, expansion and development of the role were introduced as a tool to facilitate decision making, in terms of levels of practice, and to reduce conceptual confusion among nursing professionals. Nevertheless, many years have passed since the problem was first tackled and it has yet to be fully resolved.

Valid, reliable instruments are available for assessing advanced competencies and have been employed on several occasions in Spain [25,26,27], but in general these studies do not take into account all types of nursing professionals and tend to focus on specific profiles.

**Figure 1:**
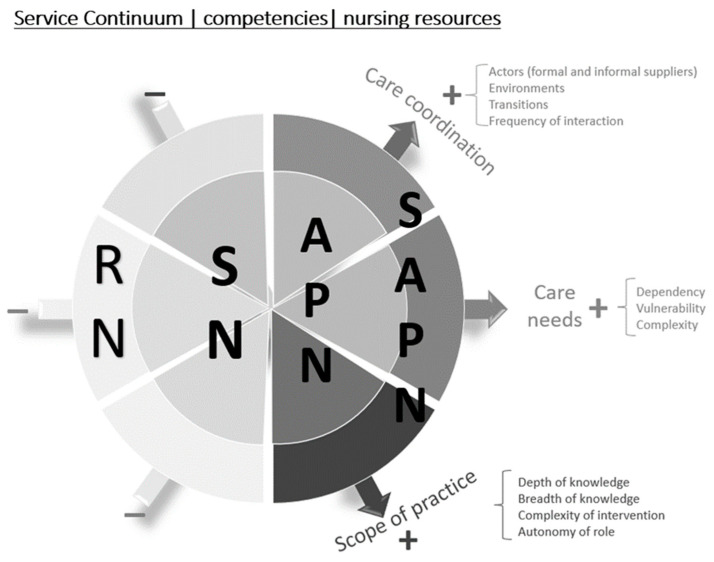
Axes for the definition of services and competencies of registered, specialist and advanced practice nurses [25]. Published with permission of the Publisher. Original Source: Gutiérrez-Rodríguez, L.; García Mayor, S.; Cuesta Lozano, D.; Burgos-Fuentes, E.; Rodríguez-Gómez, S.; Sastre-Fullana, P. et al. Competencias en enfermeras Especialistas y en Enfermeras de Práctica Avanzada. Enferm Clin. 2019;29:328-335. Copyright © 2019 Elsevier España, S.L.U. All rights reserved.

In addition to the possible coexistence of different degrees of harmonised competencies with respect to the services provided, other elements must be considered, such as the complexity of the patients treated, or the need to coordinate providers and settings. All of these factors may influence the development of specialised and advanced nursing services. In this respect, our research group [25] proposed a triple-axis model of care, considering these factors: patient care needs (marked by different degrees of dependency and vulnerability, that could lead into chronicity and the possible coexistence of multimorbidity), healthcare coordination (diverse agents providing services simultaneously, transitions between levels, frequency of interactions, settings in which care is provided) and scope of practice (depth and breadth of professional knowledge, complexity of the service to be provided and degree of autonomy in decision making) (Figure 1). The arrangement of these axes generates gradients which, depending on their combination, establish different spaces for the organisation of services provided by RNs, SNs and APNs, and even specialist advanced practice nurses (SAPNs). This model tries to explain how nursing staff should be organised on the basis of their professional profile, taking into account these three axes.

Therefore, the analysis of the distribution of competencies and the complexity of patients together can help to assess how this gradient is currently distributed, and to identify the current empirical framework, within a context in which there is only partial regulation of the practice of specialised and advanced services.

The general aim of this study is to analyse the distribution of advanced competencies among RNs, SNs and APNs in Spain. An additional, specific, objective is to determine the degree of complexity of the patients attended by these nurses, according to their professional profile.

## 2. Materials and Methods

### 2.1. Design

A cross-sectional, analytical and multicentre study was completed throughout Spain.

### 2.2. Participants

The study population consisted of nurses from the 17 regions of Spain, working in the fields of acute hospital care, out-of-hospital emergency care, primary care, residential nursing care and mental health. RNs, SNs and APNs working in any of these care settings were invited to participate in the study with the collaboration of Regional Health Care Ministries and Professional Organizations (such as Spanish Midwifery National Association, or the Spanish Mental Health Nursing Association). APNs were included if their role was formally defined by their health services, such as CMN, oncology APN, APN for complex chronic wounds, APN for diabetes or APN for ostomy and nursing. The sample was selected by convenience sampling. Since there is no formal registry of APNs in Spain, sample size was estimated form the general RN figures obtained from the Spanish National Institute of Statistics [28]. Thus, for a global population of 316,000 RNs, for *p* = q = 0.5, with a precision of 2.75%, 1265 nurses were necessary. This number was increased to cover 40% of possible missing responses.

### 2.3. Study Variables

The sociodemographic variables and those related to the participants’ professional profile included gender, age, region, years of experience, practice setting and academic training. For the SNs, the following specific variables were also collected: type of speciality, years practising as a specialist, and whether they were currently working as a SN (since, as mentioned above, in Spain SNs do not necessarily practise their speciality). The APNs were asked, in addition, how many years they had been working in their position, their job title, whether they were currently working as an APN and whether there was formal recognition of their advanced practice position in their healthcare institution.

Due to the absence of a regulatory framework for advanced nursing practice in Spain, the research team assessed the characteristics of the participants’ responses in relation to the title of the APN position held. Thus, professionals who self-declared themselves as APNs were asked to detail the title and characteristics of their position (types of patients attended, types of interventions and context of service provision). In some cases, the participants defined as APN-related tasks roles that were not in fact specific to this professional category (for example, referring to conventional clinical practice in hospitalisation units. Others had postgraduate training in areas not necessarily associated with their current work). In response to these situations, the research team agreed upon the following criteria in order to exclude erroneous self-classification as an APN:

- When the APN role is not formally recognised in the healthcare institution concerned, or where the nurse does not exercise autonomy in care practice or have decision-making capacity on advanced clinical aspects.
- When there is confusion between the performance of management functions, or between the level of professional accreditation awarded and actual advanced practice.
- When the participant uses the term “Advanced practice” but within the Spanish system is actually employed as a SN, without this service being formally classed as APN.
- When the nurse does not provide direct clinical care to a defined target population.
- In palliative care, nursing activity is only considered advanced practice if it is recognised as that corresponding to an APN role by the institution to which the nurse is affiliated.

The Advanced Practice Nursing Competency Assessment Instrument (APNCAI), which has been validated for application in Spain [26], was used to assess the advanced competency level.

Finally, the nurses were asked to rate the degree of complexity of the patients or population they had attended in their clinical practice during the last twelve months. Their answers were recorded on a Likert scale from 1 to 9, with 1 representing the minimum level of complexity, and 9 the maximum.

### 2.4. Data Collection

Data collection was conducted from June 2019 to February 2021. Nurses were invited to participate in the study via an email invitation that gave them access to an online questionnaire through the LimeSurvey platform. Prior contact was made with the heads of the health care services in the different regions, and the presidents of the nursing specialties associations to request access to the email contact details of the professionals. Invitations were sent out several times until the desired response rate was achieved.

As mentioned in the previous section, data collection in relation to nursing competencies was carried out using the APNCAI tool, which is a self-administered questionnaire with a total amount of 44 items scored on a five-point Likert-type scale ranging from “never” to “always”. The minimum score is 44 points and the maximum is 220, and it has no cut off point. APN core competencies are classified into the following dimensions: Research and Evidence-Based Practice (EBP), Clinical Leadership and Consultancy, Autonomy for Professional Practice, Interprofessional Relations and Mentoring, Quality Management, Care Management, Teaching and Professional Education and Health Promotion. This scale has a high degree of consistency, with a reliability of 0.96 measured by Cronbach’s α. In addition, the α coefficient scores for all dimensions were always above 0.80. Construct validity was also evaluated by confirmatory factor analysis.

### 2.5. Ethical Considerations

The study was approved and authorised by the Málaga Provincial Ethics Committee, and the precepts of the Declaration of Helsinki were followed. The responses to the questionnaire were anonymous, did not contain personal data and only data strictly related to the measurement of the study variables were collected. At the beginning of the online application, participants were asked to state their willingness to participate in the survey and were informed of the anonymity of their responses.

### 2.6. Data Analysis

An exploratory analysis of the sample was carried out with measures of central tendency (median, mean), dispersion (interquartile range [IQR], standard deviation [SD]) and frequency. Normality of distributions was assessed using the Kolmogorov–Smirnov test.

To assess the differences in levels of competence between RNs, SNs and APNs, the sample was first tested for homoscedasticity using the homogeneity of variances test (Levene’s test) and ANOVA. The Brown-Forsythe’s test was used instead of Levene’s test if variables were not normally distributed. Post hoc comparisons were evaluated by using the Bonferroni’s test, unless the distributions had unequal variances. In such case, Games-Howell test was used. Moreover, if normality and homoscedasticity were not granted, additional analyses were carried out by using the Kruskal-Wallis test. Finally, a Generalized Linear Model with gamma function was generated to evaluate interactions among the perceived advanced competencies of the RNs, SNs and APNs according to the complexity of the patients attended during the last twelve months. All analyses were carried out using SPSS v.25 software [29] and JAMOVI 2.3.9 [30].

## 3. Results

**Figure 2:**
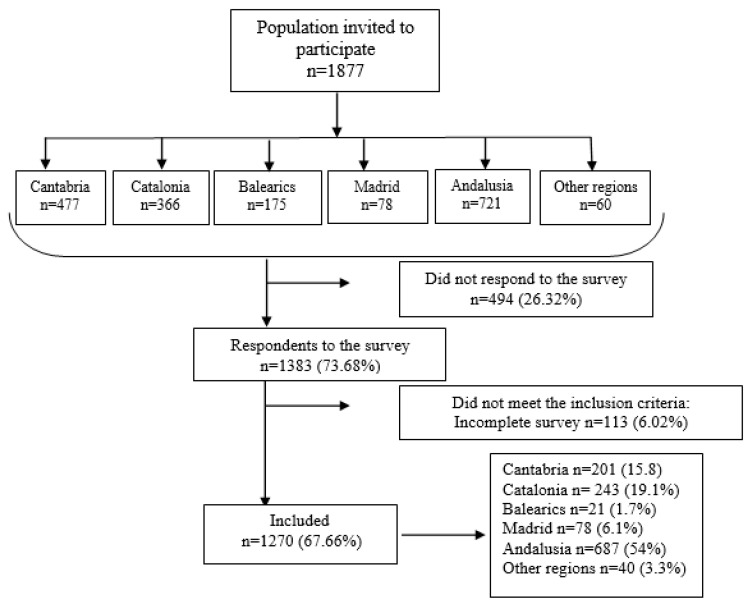
Participants’ flowchart.

The survey was sent to a total of 1877 nurses and was completed by 1270, producing a response rate of 67.66% (Figure 2). The sample was distributed throughout Spain, with the two most populated regions of Spain providing the highest absolute levels of response; Andalusia accounted for 54% of all responses, followed by Catalonia with 19.2%.

Most of the participants were women (78.8%), with a median age of 46 years (IQR: 16), who had been working as nurses for 23 years (IQR: 15) and had been in their current nursing position for 9 years (IQR: 11).

In terms of academic training, all had at least a bachelor’s degree in Nursing. Moreover, 36.7% had a master’s degree and 12.7%, a Ph.D.

Regarding the professional profile of the nurses in the sample, 46.4% (*n* = 584) were RNs, 33.7% (*n* = 428) were SNs and 19.9% (*n* = 253) were APNs. The most frequent speciality among the participants was Paediatric Nursing (14.5%), followed by Midwifery (8.4%) and Mental Health Nursing (7%). The remaining 12.2% were made up of specialists in Occupational Health Nursing, Community Nursing and Geriatric Nursing. Among APNs, 16.2% were, simultaneously, APNs and SNs. 79.5% of the institutions where the APNs worked were formalised advanced practice centres, where the APN position was officially recognised. Most nurses worked in a hospital setting (55.8%), followed by those in primary health care (30.9%). The rest worked in areas such as mental health, out-of-hospital emergencies, transitional care or nursing homes.

In relation to the overall median scores obtained in the APNCAI, the dimensions of autonomy (30; IQR; 12), research and EBP (25; IQR: 11) and care management (22; IQR: 7) obtained the highest scores. Table 1 summarises the general characteristics of the sample.

The APNs consistently self-reported a higher level of competencies than the other professionals, in each of the dimensions considered. This was also the case for the total APNCAI score; thus, the APNs obtained a median score of 178 points (IQR: 32) versus a median of 155 points (IQR: 43) for all other professionals (*p* < 0.001). The analysis of the distribution of APNCAI scores among RNs, SNs and APNs showed an ascending gradient where APNs scored the highest, followed by SNs and finally RNs (*p* < 0.001) (Table 2).

**Figure 3:**
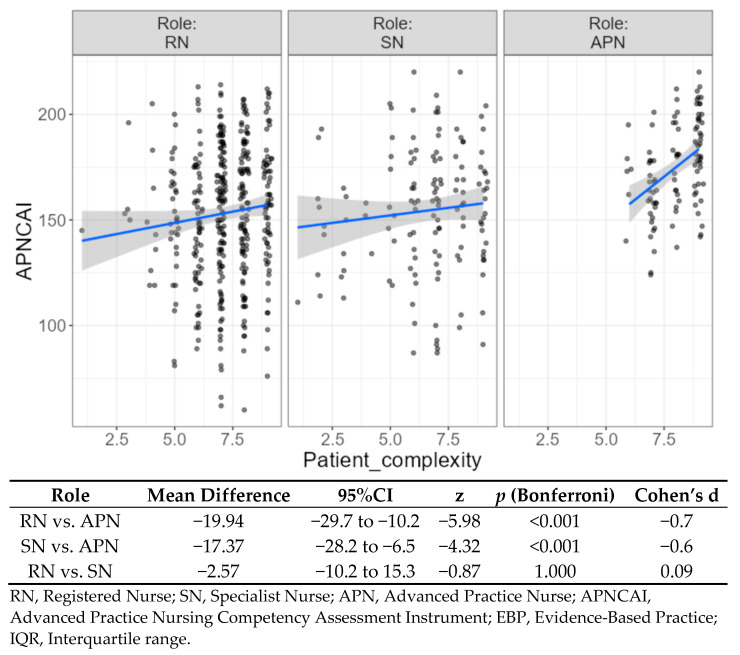
Advanced competencies among different nursing roles and complexity of patients attended by nurses.

Finally, we analysed the level of patient complexity perceived by each category of nurse during the last 12 months. The results obtained showed that patients attended by APNs presented the highest level of complexity (8 ± 2), while there were no differences in the median values obtained by SNs and RNs (7 ± 2). These results were statistically significant (*p* = 0.001). An additional analysis was carried out to evaluate the interactions among different nursing roles, the advanced competency level and the complexity of patients attended during the last 12 months. The results revealed a significant interaction between advanced competencies and patients’ complexity (*p* = 0.024). In general, APNs did not provide care to low-complexity patients, while SPs attended both patients with very low complexity and higher complexity. On the other hand, some RNs reported that they cared for high-complexity patients (Figure 3).

## 4. Discussion

The main goal of the present study is to analyse the distribution of advanced competencies in RNs, SNs and APNs in Spain and their relationship with the complexity of the patients seen.

The response rate obtained was over 66% and the female representation of the group was much higher than that of males, a very similar gender distribution to that found among nursing professionals in general in Spain, where 84.1% of nurses are women [31].

Regarding professional profiles, although a stratified random sampling was not performed, in the case of SNs a sufficient representation of all nursing specialities was obtained with the exception of Occupational Health Nursing and Geriatric Nursing, according to the strata division of Spanish nursing specialists [32]. This under-representation might have occurred because these two specialities correspond to those for which fewest places are offered annually for training as residents.

The differences in the median APNCAI scores obtained are reflected by corresponding differences in the competency gradient between APNs, SNs and RNs, with APNs having the highest level of self-perceived competencies in all dimensions, especially in the dimensions of research and EBP, autonomy, clinical leadership and consultancy and care management. These results are in line with previous research carried out in Spain [25,26,27].

It is noteworthy that leadership is the primary competency dimension in APNs, standing out above all other roles. Competency in this respect seems to be essential to the proper development and implementation of the other roles [12]. Implicit in leadership capacity is the notion of competence in clinical practice, both that of APNs and of their co-workers [33,34]. Nevertheless, without support from managers of healthcare organisations, from other healthcare professionals and even from the nurses themselves, APNs will not be able to exercise their leadership and thus fulfil their responsibilities [35,36].

Another major barrier to the optimal development of advanced practice is the lack of clarity and understanding of the APN role among nurses. The review conducted by Torrens et al. [37] noted that one of the main difficulties in implementing advanced practice roles was the lack of knowledge in this respect among the health professionals themselves. The latter finding is reflected in our own study through the participants’ answers to the question on the self-description of the APN position. This variable had to be recoded on 72 occasions, as it was often confused with management positions, professional career levels or SN roles. In Spain, moreover, the appearance and consolidation of the roles of advanced practice and nursing specialities coincide in space and time, further favouring confusion between the two roles [18].

With respect to scores for self-perceived competencies, there were no statistically significant differences between the SNs and the RNs. These results are not consistent with the definition proposed by the ICN [7], from which it could be deduced that if professionals are qualified to apply advanced knowledge in a specific branch of nursing, they should possess a higher degree of competence than one without additional specialisation. However, this could be a consequence of the particular situation in Spain, where SNs, despite having specialised training, may work in general nursing positions, and never carry out the role of specialist: the impossibility of exercising the skills acquired during their training process could influence the lack of development of these competencies. However, this supposition could not be corroborated by the data obtained in the present study, as almost half of the SNs who participated did not answer this question.

As mentioned above, in Spain although there is a system of formal specialised training, this does not necessarily translate into the formal regulation of practice at different levels: on the other hand, the professional career does have formal recognition and there are regions where there is even a system of accreditation at the professional level, as is the case in Andalusia, the largest region in Spain [38], although even here the potential of these accreditation systems is not fully exploited. Therefore, the fact that nurses are accredited at a higher level does not mean that they will care for more complex patients; rather, this “recognition” is only a progression in remuneration, but not in responsibility, functions, or the depth and scope of nursing practice.

The above picture is corroborated by the results obtained from our analysis of the interaction of the competency gradient with the treatment complexity of the patients attended. According to the participants’ responses, patients with the highest degree of complexity were mainly cared for by APNs, followed by RNs and SNs. These findings represent above all the figure of the CMN; these nurses exercise the most widespread and highly developed advanced practice roles in Spain [39] and are often responsible for the care of patients with complex chronic pathologies [6,40].

The results of our analysis suggest that the Spanish health services are not exploiting the full potential of the advanced competencies of APNs and SNs. For example, if SNs are not called upon to work in clinical settings for which they have specialised, but merely as RNs, this could heighten their perception of a lack of advanced competencies acquired during the SN training programme. According to prior research, a higher level of nursing competencies is associated with significant increases in clinical safety and the avoidance of adverse events such as readmission, morbidity and death [41]. The indiscriminate management of these competencies generates costs in terms of clinical safety and produces avoidable expense for the health service.

Some countries such as the UK have already implemented these roles with a positive impact in terms of patient and family clinical outcomes, quality of care, use of the healthcare system and its costs, as well as some aspects related to the work environment. In a scoping review carried out in the English context, it emerged that the factors that facilitate the implementation of these roles are those related to the standardisation of the education and training of these professionals, knowledge, and clarity of the functions they perform, encouraging the continuous evaluation of these functions and establishing a structured and clear professional career path [42].

On the other hand, in Switzerland, Bryant-Lukosius [43] et al. created a conceptual framework with the aim of assessing advanced practice roles within three stages that shape the development of the role: Introduction phase, implementation phase and long-term sustainability phase. In this study, they set out which objectives should be pursued in each of the stages in order to enable the proper development of these roles. Despite the complexity of the Spanish model, associated with the existence of 17 different health systems, these approaches offered by other countries could be taken as a guide to facilitate the implementation of advanced practice in Spain.

The overriding question that arises from our analysis of the gradients of competencies in Spain, the deregulation of the services offered and the systems of access and accreditation, is the extent to which decision-makers and managers take into account the impact of certain advanced services on patient outcomes, in view of the above-described context of confusion. This situation has, in fact, been apparent for decades, but without a clear commitment to high-quality, formal education for nurses, the real advances made by the nursing profession might fail to achieve the desired results [44].

The results obtained in this study enable us to more clearly depict the competencies of RNs, SNs and APNs and the service spaces they occupy, in accordance with two of the axes proposed in our model [25], namely the competency gradient of the nurses and the care needs of the populations they attend. This could help to discern which duties as well as which units and services would be most suitable for RNs, SNs and APNs to carry out their functions. The third axis, concerning the care coordination needs of the patients attended, could not be assessed in the present study, and will be the subject of future analysis.

There is a need to establish a regulatory framework for clinical practice in advanced and specialised services linked to academic and/or specialisation level, with a clearly differentiated portfolio of services and functions and with remuneration and accountability implications.

### Limitations

The main limitation of this study is its non-generalisability: due to the specificity of professional nursing regulations in Spain, the results presented cannot readily be extrapolated to other countries. Nevertheless, we highlight the consequences of the absence of a regulatory framework for APN and nursing specialties, and reveal the inefficiency incurred when health services and academic institutions invest in the development of competencies that are not subsequently implemented. Very probably, some of these factors, or similar expressions, can also be found elsewhere. Consequently, our findings could help decision-makers who are considering implementing a system of nursing specialities and APN services comparable to the Spanish experience.

On the other hand, this study could contribute to reducing the disparity of criteria among countries with similar national health care services, with respect to evaluating the scope of practice of SNs and APNs. The development of a common regulatory framework could facilitate the international mobility of nurses with high competency levels, benefiting both the nurses and their employers.

Another limitation of this study is the low response rate obtained for some of the variables in the questionnaire, such as the nurses’ academic degree. With sufficient representativity, other interesting analyses of our sample could have been conducted with respect to academic training, the influence of the possession of a master’s degree in the case of APNs, etc. However, due to the low statistical power in this respect, these analyses could not be carried out.

Future studies should be undertaken to further consider the influence of the working environment on nursing roles, especially concerning the development of advanced competencies.

## 5. Conclusions

This study reveals differences in levels of self-perceived competencies among RNs, SNs and APNs, with the latter group presenting the highest level in all dimensions of advanced competencies, followed by SNs and RNs. In terms of the complexity of the patients attended, APNs most commonly provide care to the most complex patients, while SNs are not necessarily dedicated to caring for more complex cases.

It is crucial to establish a regulatory framework for clinical practice in advanced and specialised services, accompanied by academic training and/or specialisation, together with the harmonisation of academic and skill-mix levels, accreditation and autonomy across different nursing roles within the health care system.

## Figures and Tables

**Table 1:** Descriptive variables of the sample.

Subjects’ Characteristics		Median (IQR) or Frequency (%)
**Sex**	Female	998 (78.8)
	Male	268 (21.2)
**Age**		46 (16)
**Years of profession**		23 (15)
**Master**	Yes	285 (36.7)
	No	491 (63.3)
**PhD**	Yes	85 (12.7)
	No	582 (87.3)
**Context of practice**	Hospital care	670 (55.8)
	Primary care	371 (30.9)
	Mental Health	80 (6.7)
	Emergency	70 (5.8)
	Transitional care	5 (.4)
	Nursing homes	5 (.4)
**Nursing specialty**	No specialty	800 (66.4)
	Paediatric Nursing	175 (14.5)
	Midwifery	101 (8.4)
	Mental Health Nursing	84 (7)
	Occupational Health Nursing	22 (1.8)
	Family and Community Nursing	16 (1.3)
	Geriatric Nursing	6 (.5)
**Do you currently practice the specialty you hold?**	Yes	222 (89.9)
	No	25 (10.1)
**Advanced practice nursing**	Yes	253 (23.4)
	No	829 (76.6)
**Do you practice as advanced practice nurse?**	Yes	41 (16.7)
	No	212 (83.8)
**APNCAI score**	Research and EBP (8–40) *	25 (11)
	Leadership, consultancy (4–20)	12 (7)
	Autonomy (8–40)	30 (12)
	Relationship, mentorship (5–25)	21 (5)
	Quality management (5–25)	17 (6)
	Care management (6–30)	22 (7)
	Professional education (4–20)	19 (4)
	Health promotion (4–20)	16 (5)
	Total score (44–220)	159 (43)

IQR, Interquartile range; SN, Specialist Nurse; APN, Advanced Practice Nurse; APNCAI, Advanced Practice Nursing Competency Assessment Instrument; EBP, Evidence-Based Practice. * Minimum and maximum possible scores in each dimension.

**Table 2:** Gradient of scores between RNs, SNs and APNs.

APNCAI Dimensions	RN (*n* = 589)	SN (*n* = 428)	APN (*n* = 253)	*p*
	Median (IQR)	Median (IQR)	Median (IQR)	
**Research and EBP (8–40)**	23 (11)	24 (11)	28 (7)	<0.001
**Leadership, consultancy (4–20)**	11 (7)	12 (7)	16 (4)	<0.001
**Autonomy (8–40)**	28 (12)	29 (12)	33 (7)	<0.001
**Relationship, mentorship (5–25)**	20 (6)	20 (6)	23 (4)	<0.001
**Quality management (5–25)**	17 (7)	17 (6)	19 (5)	<0.001
**Care management (6–30)**	21 (7)	21 (7)	25 (5)	<0.001
**Professional education (4–20)**	18 (4)	19 (4)	19 (3)	<0.001
**Health promotion (4–20)**	16 (5)	16 (4.75)	19 (4)	<0.001
**Total score (44–220)**	152 (46)	155 (42)	178 (32)	<0.001

RN, Registered Nurse; SN, Specialist Nurse; APN, Advanced Practice Nurse; APNCAI, Advanced Practice Nursing Competency Assessment Instrument; EBP, Evidence-Based Practice; IQR, Interquartile range.

## Data Availability

The data presented in this study are available on request from the corresponding author.
